# Characterization of a primary cellular airway model for inhalative drug delivery in comparison with the established permanent cell lines CaLu3 and RPMI 2650

**DOI:** 10.1007/s44164-024-00079-y

**Published:** 2024-11-25

**Authors:** Janik Martin, Rebecca Rittersberger, Simon Treitler, Patrick Kopp, Anit Ibraimi, Gabriel Koslowski, Max Sickinger, Annabelle Dabbars, Katharina Schindowski

**Affiliations:** 1 Institute of Applied Biotechnology, University of Applied Science Biberach, Hubertus-Liebrecht Strasse 35, 88400 Biberach, Germany; 2 Faculty of Natural Science, University of Ulm, Albert-Einstein-Allee 11, 89081 Ulm, Germany; 3 Justus-Von-Liebig-Schule, Von-Kilian-Straße 5, 79762 Waldshut-Tiengen, Germany

**Keywords:** Porcine, Airway mucosa, Respiratory drug delivery, Biopharmaceuticals, IgG, FcRn

## Abstract

**Purpose:**

For optimization of respiratory drug delivery, the selection of suitable in vitro cell models plays an important role in predicting the efficacy and safety of (bio)pharmaceutics and pharmaceutical formulations. Therefore, an in-depth comparison of different primary and permanent in vitro cellular airway models was performed with a focus on selecting a suitable model for inhalative antibodies.

**Methods:**

Primary cells isolated from the porcine trachea were compared with the established human cell lines CaLu3 and RPMI 2650. The in vitro models were characterized for different epithelial markers by real-time quantitative polymerase chain reaction, which provides insight into the cellular composition of each model. For a few selected markers, the results from RT-qPCR were confirmed via immunofluorescence. Barrier integrity was assessed by transepithelial electrical resistance measurements and FITC-dextran permeability.

**Results:**

Primary cell models retain key features of the respiratory epithelium, e.g., the formation of a tight epithelial barrier, mucin production, and the presence of club/basal cells. Furthermore, the expression of Fc receptors in the primary cell models closely resembles that in respiratory mucosal tissue, an essential parameter to consider when developing therapeutic antibodies for inhalation.

**Conclusion:**

The study underlines the importance of selecting wisely appropriate in vitro models. Despite the greater effort and variability in cultivating primary airway cells, they are far superior to permanent cells and a suitable model for drug development.

**Supplementary Information:**

The online version contains supplementary material available at 10.1007/s44164-024-00079-y.

## Introduction

Inhalative drug delivery of small molecules has been an established application method for decades, providing an efficient treatment for the therapy of, e.g., asthma or chronic obstructive pulmonary disease. With regard to biopharmaceuticals, until a few years ago, the market appeared quite sparse apart from dornase alfa, fluctuating insulin products, and the off-label use of inhaled heparin [1]. With the beginning of the SARS-CoV-2 pandemic, targeting the respiratory tract gained increasing interest for the application of biopharmaceuticals, nucleic acid, and virus-based vaccines. According to clinicaltrials.gov, search results for the terms “inhalation” AND “antibody” increased by 50% in 2020–2024 compared to 2016–2020 (the search was conducted on 15 May 2024) [2, 3]. But the reasons underlying this trend are not only due to the pandemic, as the inhalative route represents the most direct and local approach, bypassing a rapid elimination through the first-pass effect, and thus is likely to result in fewer side effects. Nevertheless, inhalation can also be used for systemic treatments as the respiratory tract has a high surface area of 80–120 m^2^, high blood supply, and a thin barrier between alveoli and blood capillaries. Thus, the respiratory tract also presents an optimal absorption side [1, 4–6]. When developing inhalable (bio-)pharmaceutics, suitable in vitro models are crucial, which mimic optimally the airway tissue. Several cell-based in vitro models have been developed to simulate the airway environment for drug delivery. These include immortalized cell lines such as CaLu3 and RPMI 2650, which are widely used in the context of airway modelling, as well as more advanced primary cell cultures that closely resemble the airway tissue in terms of cellular diversity and functionality [7–12]. Several immortalized cell line models do not mimic the complex multicellular and functional properties of the airway epithelium, particularly under air–liquid interface (ALI) conditions [13]. For example, CaLu3 and RPMI 2650 offer practical advantages for large-scale studies, but they are limited in their differentiation potential and have altered metabolic profiles. This highlights the need for more physiologically relevant models, like primary cell models. Comparisons of different cell-based models in the literature show that primary cells, although more complex to handle, generally offer higher predictive power for in vivo outcomes [13–15].

Regarding the anatomical structure of the respiratory tract, inhaled drugs are deposited in the upper airways, such as the mouth and oropharynx, as well as in the lower airways, including the trachea, bronchi, and lungs. Several parameters determine the deposition pattern of an inhaled biopharmaceutical, like the particle or droplet size, the mode of inhalation, the breathing pattern of the patient, and the degree of blockage in the airways [5]. On a cellular level, the mucociliary clearance caused by beating cilia lined up on the conductive airways and cough clearance represents the first obstacle that needs to be overcome [6]. The next barrier to the outside world is represented by the respiratory epithelium, a pseudostratified cell layer consisting of (ciliated) columnar epithelial cells, goblet cells, club cells, and basal cells [16]. With tight and adherent junctions, the epithelium forms a strong and stable barrier to shield the submucosa against the outer world; it produces and propels the mucus and secretes antimicrobial peptides to prevent invasion of pathogens and other particles [17].

The paracellular transport is suitable for molecules with a radius smaller than 11 Å [18]. Therefore, biopharmaceuticals, especially immunoglobulin G (IgG), are mainly transported transcellularily through the “neonatal” Fc fragment of IgG receptor and transporter (FcRn) [19]. FcRn is an important receptor for transcytosis, recycling, and the rescue of IgG from lysosomal degradation in endothelial cells, epithelial cells, and monocytes. Transcytosis can occur in both directions: from the apical to the basolateral side, and vice versa, but also recycling to the same side is well described for FcRn [13, 14]. But not only the epithelial cells are relevant for IgG uptake via the airways, also the *lamina propria* underneath the epithelium is a structural compartment that needs to be considered. This connective tissue contains blood and lymphatic vessels, glands, elastic fibers, and, depending on the localization in the respiratory system, various immune cells to prevent progression of infection by invading pathogens [20, 21]. An important group of receptors on immune cells is the Fc fragment of IgG receptors (FCGR). These receptors mostly bind immune complexes of IgG with antigens but also monomeric IgG. In addition to immune cells, other cell/tissue types throughout the body have also been described to express FCGR as well [22]. For example, FCGRIIb has been shown to be the responsible receptor for the transcytosis of IgG through the placenta and has also been discussed to play a part in the transport of applied IgG in the olfactory mucosa for central nervous system (CNS) delivery [8, 13, 14, 23]. However, it needs to be further investigated if also other receptors play a role in mucosal transport.

For the development of new inhalable (bio-)pharmaceutical drugs such as therapeutic IgGs, suitable in vitro models with a high predictivity for in vivo and clinical translation are essential for the preclinical phase. While several models exist, there is a growing need for more comparative studies to evaluate the performance of different in vitro systems, particularly those using primary cells versus permanent cell lines, for their application in inhalation studies. The most commonly used permanent cell lines consist of one cell type only and can differentiate only to a limited extent even under air–liquid interface (ALI) conditions [12]. Additionally, functional and genetic alterations further decrease their relevance for later in vivo studies [19, 24]. In the present study, the cell lines CaLu3 and RPMI 2650 were used as reference models. CaLu3 are described to form a tight barrier and produce mucins when cultivated under ALI conditions [25, 26]. RPMI 2650 have been reported to form multi-layered, leaky barriers and to have an impaired metabolic activity [11, 12]. In contrast to cell lines, primary cells more closely resemble the original airway tissue and consist of various cell types. During their isolation and purification, epithelial progenitor and basal cells are obtained. These cells are essential for proliferating into a confluent cell layer and differentiating into a suitable in vitro epithelium model that exhibits the characteristics of the different cell types described above [27].

As human respiratory tissue is not always available, is rather expensive, and also difficult for ethical reasons, the use of pig (*Sus scrofa*) airway mucosa as a promising alternative for the extraction of trachea mucosal primary cells (TMPC) was demonstrated and characterized here. The histological and cellular composition of the respiratory mucosa in pigs is comparable to humans’ [28, 29]. Furthermore, the porcine immune system shows over 80% similarity to the human one [30], and it could be shown that human IgG is transported by porcine FcRn into the porcine olfactory mucosa [31]. Additionally, the porcine trachea is comparable in size to humans’ and provides a suitable amount of tissue for cell extraction [32]. Therefore, significantly more primary cells can be extracted from pigs than from mice due to their larger airways. For the characterization and evaluation of the primary cell line models, cells were cultivated under ALI conditions to mimic direct contact to the air as in the respiratory system [33].

The aim of this work was to establish and in-depth characterize porcine primary cell models of the conducting airways, compare it to two commonly used permanent airway cell models, CaLu3 and RPMI 2650, and then to consider the in vitro models’ suitability for drug delivery studies with (bio-)pharmaceuticals, in particular IgG.

## Materials and methods

### Cell culture

All different cell models were cultured in transwell inserts (ThinCert™ for 24-well plate, pore size 1 µm, transparent, Greiner Bio-one, Frickenhausen, Germany) with a basolateral volume of 260 µL cell culture medium as indicated below for each cell model. After seeding into transwell inserts, cells were cultured for 24 h under submerged condition with 100 µL culture medium added to the apical compartment. Then, the apical medium was removed to airlift the cells and expose them to ALI for 21 days. Three times per week, the apical side of the cells was washed with 200 µL PBS, and the culture medium in the basolateral compartment was exchanged with 260 µL fresh medium during this 21-day period.

#### Tracheal mucosal primary cells (TMPC)

**Fig. 1:**
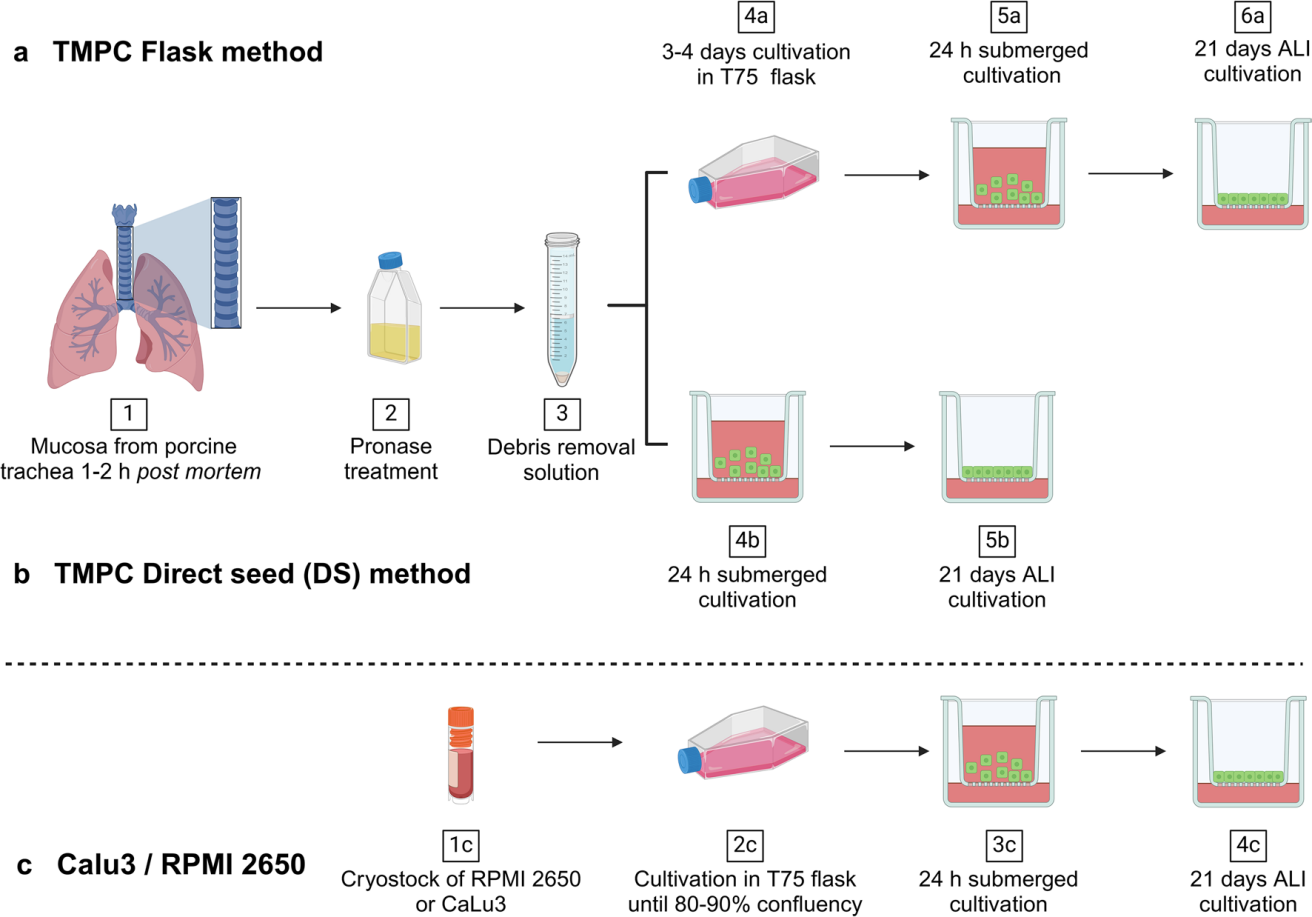
Workflow of cell cultivation procedures of the different in vitro models: For the extraction of TMPC, the mucosa was peeled off the cartilage of a porcine trachea (1), digested in Pronase (2), and separated from debris by a gradient centrifugation (3). While TMPC Flask were first expanded under submerged conditions in a T75 flask for 3 to 4 days (4a) before seeding them into membrane inserts (5a), TMPC DS were directly seeded into membrane inserts (4b). Both models were subjected to an airlift after 24 h and allowed to differentiate at air–liquid interface (ALI) for 21 days (6a and 5b). The permanent cell lines CaLu3 and RPMI 2650 were cultivated from cryostocks (1c, 2c) in flasks prior to cultivation in membrane inserts (3c). The cells were airlifted after 24 h and allowed to differentiate to an epithelial barrier model for 21 days (4c). Sketch created under license with BioRender

TMPC were extracted within 1–2 h *post mortem* from the tracheal mucosa of 4- to 6-month-old pigs from a local slaughterhouse as depicted in Fig. 1a(1). First, the tracheal mucosa was removed from the hyaline cartilage. Subsequently, the tissue samples were disinfected using Octenisept® (Schülke & Mayr GmbH, Norderstedt, Germany) and were washed twice with prewarmed PBS. After that, the samples were transferred into T25 flasks, 20 mL Pronase medium (1.4 mg/mL Pronase (Sigma-Aldrich, Taufkirchen, Germany)) was added, and the flask was incubated at 37 °C, 5% CO_2_, and 95% rH for 1 h (Fig. 1a(2)). The supernatant containing TMPC was removed and processed with debris removal solution (Miltenyi Biotec, Bergisch Gladbach, Germany) according to manufacturer’s instructions (Fig. 1a(3)). From this point, two different cultivation methods were performed: the “Flask method” (TMPC Flask; Fig. 1a) and the “Direct seed method” (TMPC DS; Fig. 1b). Coating of all surfaces (flasks or membrane inserts) was performed with 0.05 mg/mL rat tail collagen type I (Primacyte, Schwerin, Germany) solution at least 2 days before cultivation.

For TMPC Flask (Fig. 1a), the cells were processed with the debris removal solution, the supernatant was discarded, and the cell pellet was resuspended in adequate volumes of primary cell culture cultivation medium (DMEM: F12 (1:1), 20% FBS, 2 mM glutamine, 1% NEAA, 5 µg/mL amphotericin B, 200 µg/mL gentamycin sulfate and kanamycin, 20 U/mL penicillin, 20 µg/mL streptomycin, and 4.5 g/L Glu). The resuspended cells were cultivated at 10^6^ cells/mL in coated T75 flasks at 37 °C, 5% CO_2_, and 95% rH (Fig. 1a(4a)). Cells were allowed to adhere for 4 h, and the primary cell culture cultivation medium was removed. Cells were washed with adequate volumes of PBS, and fresh primary cell culture cultivation medium was added. When cells reached 80 to 100% confluency (± 4 days after cultivation), potential contaminating fibroblasts were removed by incubation of the cell layer with a trypsin/EDTA solution. The fibroblast-containing trypsin/EDTA solution was discarded. The remaining epithelial cells were detached after an additional incubation with 1 volume of trypsin/EDTA, the addition of 1 volume cultivation medium, and with the aid of a cell scraper. The cells were seeded into membrane inserts at a cell density of 10^5^ cells/insert (Fig. 1a(5a)). Cells were airlifted after 24 h under submerged conditions and allowed to differentiate at the ALI for 21 days (Fig. 1a(6a)).

The direct seed (DS) method represents a faster way to culture TMPC in membrane inserts. After isolating the cells with Pronase digest and density gradient centrifugation using the debris removal solution, cells were directly seeded into membrane inserts with a cell density of 10^5^ cells/insert (Fig. 1b(4b)) with the same culture medium as for the TMPC Flask model. Like in the TMPC Flask procedure, the cells were airlifted after 24 h under submerged conditions and allowed to differentiate at the ALI for 21 days (Fig. 1b(5b)).

#### RPMI 2650

RPMI 2650 cells were thawed from cryostocks (Fig. 1c(1c)) and cultivated in MEM, 10% FBS, 2 mM glutamine (Gibco®, Invitrogen, Darmstadt, Germany) in T-flasks at 37 °C, 5% CO_2_, and 95% rH until they reached 80–90% confluency (Fig. 1c(2c)). Cells were washed twice with prewarmed PBS and were detached by trypsinization using trypsin/EDTA solution (Gibco®, Invitrogen, Darmstadt, Germany). RPMI 2650 cells were seeded into membrane inserts (Fig. 1c(3c)) with a cell density of 10^5^ cells/insert in MEM, 10% FBS, 2 mM glutamine supplemented with 10 U/mL penicillin and 10 µg/mL streptomycin (Gibco®, Invitrogen, Darmstadt, Germany). Cells were airlifted after 24 h under submerged conditions and allowed to differentiate at the ALI for 21 days (Fig. 1c(4c)).

#### CaLu3

CaLu3 cells were thawed from cryostocks (Fig. 1c(1c)) and cultivated in DMEM:F12 (1:1), 15% FBS (Capricorn, Ebsdorfergrund, Germany) in T-flasks at 37 °C, 5% CO_2_, and 95% rH until they reached 80–90% confluency (Fig. 1c(2c)). For seeding CaLu3 in inserts, cells were washed twice with prewarmed PBS and were detached by trypsinization with trypsin/EDTA solution (Gibco®, Invitrogen, Darmstadt, Germany). CaLu3 cells were seeded into membrane inserts with a cell density of 10^5^ cells/insert (ThinCert™ 1 µm, transparent, Greiner Bio-one, Frickenhausen, Germany; Fig. 1c(3c)) in DMEM:F12 (1:1), 15% FBS supplemented with 10 U/mL penicillin and 10 µg/mL streptomycin. Cells were airlifted after 24 h under submerged conditions and allowed to differentiate at the ALI for 21 days (Fig. 1c(4c)).

### TEER

To determine TEER, the cultivation medium was removed from the membrane inserts, and 350 µL MEM w/o phenol red (Gibco®, Invitrogen, Darmstadt, Germany) was added apically, and 500 µL MEM w/o phenol red was added to the basolateral compartment. Inserts were placed in the incubator for 10 min at 37 °C, 5% CO_2_, and 95% rH, followed by another 10-min incubation at room temperature to let cells equilibrate. The electrical resistance was measured using an EVOM3 device with chopstick electrodes (World Precision Instruments, Sarasota, FL, USA). The blank was subtracted from the raw data and multiplied with the area of the membrane inserts (0.336 cm^2^).

### FITC-dextran permeation and P_app_ value

In addition to the TEER, the barrier function of the cells was characterized by fluorescein isothiocyanate-dextran (FITC-dextran) 4 kDa permeability by replacing the basolateral medium with 260 µL MEM medium w/o phenol red and applying 100 µL 500 µg/mL FITC-dextran (Sigma-Aldrich, Taufkirchen, Germany) in PBS to the apical/luminal compartment. After 0.5 h, 1 h, 2 h, 4 h, 8 h, and 24 h, 20 µL samples were removed from the basolateral compartment and supplemented with 20 µL MEM w/o phenol red after each sampling to keep the volume constant. FITC-dextran was quantified in a Tecan Infinite 200 Pro (Tecan, Männedorf, Switzerland) at 490 nm/520 nm. The apparent permeability coefficient (*P*_app_) was calculated according to Eq. (1):

1 $${P}_{\mathrm{app}}\left(\frac{\mathrm{cm}}{\mathrm{s}}\right)= \frac{{\Delta \left[c\right]}_{\mathrm{b}} \times { V}_{\mathrm{b}}}{A\times {[c]}_{\mathrm{a}} \times\Delta t}$$

Δ[*c*]_b_: basolateral concentration (µg/mL)

[*c*]_a_: apical concentration (µg/mL)

Δ*t*: time of measurement (s)

*A*: growth area of the membrane (cm^2^)

*V*_b_: basolateral volume (mL)

### Real-time quantitative PCR (RT-qPCR)

Total mRNA was extracted with RNeasy Plus Mini Kit (QIAGEN, Hilden, Germany); cDNA synthesis was performed with LunaScript RT SuperMix (New England Biolabs, Frankfurt am Main, Germany), and the remaining gDNA was digested using gDNA Removal Kit (Jena Bioscience, Jena, Germany). Luna Universal qPCR Master Mix (New England Biolabs, Frankfurt am Main, Germany) was used for RT-qPCR in a LightCycler (Roche, Mannheim, Germany). All methods were performed according to manufacturer’s instructions.

Results are given as ∆*c*_p_ and were calculated based on Eq. (2).

2 $$\Delta {c}_{{\mathrm{p}}_{\mathrm{sample}}}= {c}_{{\mathrm{p}}_{\mathrm{sample}}}- {c}_{{\mathrm{p}}_{\mathrm{GAPDH}}}$$

Primer design was done using the NCBI Primer design tool “Primer-BLAST.” Glycerinaldehyd-3-phosphat-dehydrogenase (GAPDH) was used as housekeeping gene reference. Tables 1 and 2 show the primer sequences and targets used in RT-qPCR.

**Table 1:** RT-qPCR primer sequences of different markers for the porcine airway mucosa

Marker for	mRNA target	Forward primer (5′-3′)	Reverse primer (5′-3′)
Housekeeping	*GAPDH*	GTTGTGGATCTGACCTGCCG	CAGCCCCAGCATCAAAGGTAG
Tight junctions	*OCLN*	TTAAAAACGTGTCGGCAGGC	GCATAGTCCGAAAGGGGAGG
	*CDH1*	TTCAAGAAGCTGGCGGACAT	AGTCCCCTAGTCGTCCTCAC
Ciliated cells	*FOXJ1*	CTTCCAGAACCTTCCTTTGGC	TCGAATGTGGTGTCTGGCTCA
	*TUBB4B*	CCGCTCTTCGGCTTTTCTTAC	CGCTGATCACCTCCCAAAACTTG
	*CETN2*	TCGAATGTGGTGTCTGGCTCA	AGTGCCCTCATTGCCACCTTA
Airway epithelial cells	*MUC1*	AAGATCCCACCACCAGCTAC	GCTAAGGTTTGATGCAAGGGG
	*MUC4*	TCACACCACCACTTCAGTCC	TGTCATAGTGTTTCCACCCAGG
Fibroblasts	*COL3A1*	CCGAGCTTCCCAGAACATCA	CCCCAGTGTGTTTAGTGCAAC
	*LUM*	GGCTCCTTTGATGGACTGGT	GCCAGAAGGCAGTTTGGTCA
Basal cells	*PDPN*	ACTGTAGGAAGCACAACGCA	CCGTCCCCAAGCCATCTTT
	*KRT5*	GGCGAGGAGTGCAGATTGAG	GGTGTTTGTGACGACCGAGA
Goblet cells	*MUC5AC*	CTGACCAAGAGCTCCGTCCT	CGCAGGTCTTGTTGGCGTAT
	*MUC5B*	CACCCACATCGTCCTCAAGA	TGTCCTCTCCGTTCCACACA
Club cells	*SCGB1A1*	GACAGTGTGTTAAAACTCCAGGAAA	TCAGGGCCTCCAGAGATGAA
	*SCGB3A2*	GGCATTTCTGTTGAGCACCTG	CAAGTATGAGAGAGCCTCCAGC
Fc-receptors	*FCGRT*	GAGAGTGACATCGGCCCCAA	CCAACCACTGGCATGGAGGA
	*FCGR1A*	CATGGGAAGCAAGACCCTGA	CTGCGCTTGATGACCTTTCC
	*FCGR2A*	GCAAAGTTGAGGAGTTGGGGG	TGAACCCAGTGAGATTGGGCA
	*FCGR2B*	GGAGCCCATCTTGCTGCGGT	AGCTGGACCCTTGGACAGTG
	*FCGR3A*	GCTGGCACACACGCTGAAGA	CCCTGGCACTTCAGAGTCACA

**Table 2:** RT-qPCR primer sequences of different markers for the human airway mucosa

Marker for	mRNA target	Forward primer (5′-3′)	Reverse primer (5′-3′)
Housekeeping	*GAPDH*	GGTCGGAGTCAACGGATTTGG	TTCTCAGCCTTGACGGTGCC
Tight junctions	*OCLN*	CCCAGTTGCGGCGAGCGGATTG	AGAGGCCTGGATGACATGGCTGA
	*CDH1*	ACCACTGGGCTGGACCGAGAGAGTT	CCAAGCCCGTGGTGGGATTGA
Ciliated cells	*FOXJ1*	GCGCTCTGAGCCAGGCACCACA	CGCGCTCTCTGGCCCGCTGA
	*TUBB4B*	TGCGCGCCCGCTCTTCTGC	GCTGATCACCTCCCAAAACTTGGCG
	*CETN2*	GCGGACTCCTTTGGCTATGGCCTC	AGCCCAGGGCCCTCATTGCCA
Airway epithelial cells	*MUC1*	TGGGGCATCGCGCTGCTGGT	GGCGGCACTGACAGACAGCCAAGG
	*MUC4*	GCGAACGCCACCCTCAATCA	TGGTCTGCCCCTTGTAGGCT
Fibroblasts	*COL3A1*	TGGTCAGCAGGGTGCAATCGGCA	GGTGGCCTGGGGAGCCCTCAGAT
	*LUM*	CTTCAATCAGATAGCCAGACTGC	AGCCAGTTCGTTGTGAGATAAAC
Basal cells	*PDPN*	CGGGAAGGTACTCGCCCTAA	GGCAAGTGTTCCACGGGTCA
	*KRT5*	GGGCGAGGAATGCAGACTCA	TGCTACCTCCGGCAAGACCT
Goblet cells	*MUC5AC*	CATCAACGGGACCCTGTACC	CTGGTACTCGAAGCCCACAG
	*MUC5B*	AAGCCCTTCCACTCGAACTG	CATCCACCACGTAGGCGTAG
Club cells	*SCGB1A1*	CAAAAGCCCAGAGAAAGCATC	CAGTTGGGGATCTTCAGCTTC
	*SCGB3A2*	CTGTGAAGAAACTGCTGGAGGCGCT	TCCATCCCCATCCACCTCCGCTCT
Fc-receptors	*FCGRT*	GGAGCGAGGCTGAAGGGAACGTCG	CGGGGCAGGCGAGGACACCG
	*FCGR1A*	TGCTCCTTTGGGTTCCAGTTGATGG	CACTGCCTTTGTGGTGTCCACTTGC
	*FCGR2A*	ACAGTTTTGCTGCTGCTGGCTTCT	GGCTGCGAGCCCCCTGGCAT
	*FCGR2B*	TGAGGCTGACAAAGTTGGGGCTGA	TCAGGCTCTTCCAGAGCATCCGGG
	*FCGR3A*	TCAGCTGGCATGCGGACTGAAGA	AGGGGAGTAGGCTCCCTGGCA

### Immunofluorescence (IF) staining

After 21-day differentiation under ALI conditions, the apical and basolateral media were removed, and membrane inserts were placed into 4% chilled paraformaldehyde (PFA) or a 1:1 mixture of ice-cold methanol and acetone for at least 10 min. For the staining, the fixative was removed, and the inserts were washed 3 × in PEMT (80 mM PIPES, 5 mM EGTA, 1 mM MgCl_2_ × 6 H_2_O, 0.2% Polysorbate 20, and 18.5 mL 10 M NaOH, pH 7.4 in 1 L MilliQ), 5 min each. Afterwards, the inserts were incubated in a quenching buffer (150 mL PEM buffer, 0.40 g NH_4_Cl) and again rinsed in PEMT. One hundred microliters of blocking buffer (0.5% BSA in PEMT) was applied to each membrane insert and incubated overnight at 4 °C or for 1 h at room temperature (RT). Next, 100 µL of antibody solution was added to the slides. Primary antibodies (Table 3) were used in a 1:100 dilution in blocking buffer and incubated overnight at 4 °C. Next, the membranes were rinsed 3 times in blocking buffer for 10 min each and incubated for 1 h at RT with 100 µL of 20 µg/mL DAPI (Thermo Fisher, Sindelfingen, Germany) and a 1:500 dilution of the secondary antibodies (Table 3) in blocking buffer. Subsequently, the inserts were washed 3 times in PEMT at 10 min each. Afterwards, the membranes were separated from the insert using pliers and mounted on slides. The slides were embedded with DABCO (Sigma-Aldrich, Taufkirchen, Germany) containing Mowiol. Analysis was performed using either a Zeiss LSM 710 (Carl Zeiss, Oberkochen, Germany) confocal microscope or a Keyence BZ-X800 (Keyence, Neu-Isenburg, Germany) fluorescence microscope. The Zeiss microscopy images were taken using either Objective Plan-Apochromat 20x/0.8 (Carl Zeiss, Oberkochen, Germany) or Objective Plan-Apochromat 40x/1.3 Oil PH3 (UV) Vis/IR (Carl Zeiss, Oberkochen, Germany). The Keyence microscopy images were taken using either Plan Apochromat 20 × BZ-PA20 (Keyence, Neu-Isenburg, Germany) or Plan Apochromat 40 × BZ-PA40 (Keyence, Neu-Isenburg, Germany).

**Table 3:** Primary and secondary antibodies used for immunofluorescence (IF)

		Host, clone	Manufacturer
Primary antibody			
Anti-FcRn^*^		Rabbit, polyclonal	Pirsbrigth institute
Anti-FcRn^**^		Rabbit, polyclonal	16190–1-AP (Proteintech)
Anti-Mucin 5AC (MUC5AC)		Mouse, monoclonal	MA5-12178 (Thermo Fisher)
Anti-Aquaporin 5 (AQP5)		Rabbit, polyclonal	PA5-36529 (Thermo Fisher)
Anti- (Cyto)Keratin 5 (KRT5)		Rabbit, polyclonal	NBP2-95276 (Novus Biologicals)
Anti-*zonula occludens-*1 (ZO-1)		Rabbit, polyclonal	40–2200 (Thermo Fisher), NBP1-85047 (Novus Biologicals)
Anti-α-acetylated tubulin		Mouse, monoclonal	T7451 (Sigma-Aldrich)
Secondary antibody			
Goat (Fab)_2_ anti-rabbit AlexaFluor 568		Goat, polyclonal	A-21069 (Thermo Fisher)
Goat (Fab)_2_ anti-rabbit AlexaFluor 488		Goat, polyclonal	A-11070 (Thermo Fisher)
Goat (Fab)_2_ anti-mouse AlexaFluor 488		Goat, polyclonal	A-11017 (Thermo Fisher)
Goat (Fab)_2_ anti-mouse AlexaFluor 647		Goat, polyclonal	A-21237 (Thermo Fisher)

*Used for IF staining on membrane inserts

**Used for IF staining on tracheal tissue

### Illustrations and statistical analysis

Graphs were created, and statistical evaluations, including either one-way or two-way ANOVA, were performed using GraphPad Prism 9 and 10 software (Boston, USA). Detailed information on statistical analysis is indicated below each graph. Microscope images were analyzed and processed with the ZEN 2.3 lite (Keyence, Neu-Isenburg, Germany) and KEYENCE BZ-X800-Analyzer 1.1.2.4 (Carl Zeiss, Oberkochen, Germany) software. Figure 3 is assembled with Microsoft® Powerpoint® 2019 (Microsoft Corporation, Redmond, USA).

## Results and discussion

The increasing interest for inhalative drug delivery requires suitable and well-characterized cellular in vitro models. Based on our previous experience with nasal primary cell models, we focused here on the lower airways and performed cell extraction from the respiratory mucosa of the trachea as previously described for nasal respiratory mucosa with cultivation in flasks (TMPC Flask; Fig. 1a) [19]. To save 3–4 days of cultivation time, a direct seed (TMPC DS) into membrane inserts was explored in parallel [34–37] (Fig. 1b). For both methods, tracheal cells were isolated and purified within a short *post-mortem* delay with material obtained from a local slaughterhouse. Both Flask and DS cultures were allowed to differentiate 21 days under ALI conditions, characterized and compared to the permanent cell lines CaLu3 and RPMI 2650 (Fig. 1c). Morphological comparison using light microscopy revealed no detectable difference between TMPC Flask and DS. Both methods resulted in a characteristic cobblestone pattern and grew in monolayers as previously described for olfactory and respiratory porcine primary cells [19]. For the TMPC Flask method, an exemplary image from the culture flask has been included in Supplementary Information 1 to display their morphology. RPMI 2650 cells in comparison grew in several overlapping multilayers and showed marginally flattened and round cells as previously shown by Salib et al*.* [38]. CaLu3 cells showed a cuboidal and polygonal morphology and grow in multilayers [39]. In general, due to the perforated membrane, it should be noted that images of cells growing in the inserts provide limited information, as they are difficult to bring into focus.

### Expression of tissue-specific epithelial markers

To ensure that the cellular airway models have the greatest possible relevance for clinical translation, they should represent the tissue that they were derived from as accurately as possible. Therefore, several markers for tight and adherens junctions, ciliated epithelial cells, airway epithelial cells, fibroblasts, basal cells, goblet cells, and club cells were investigated by RT-qPCR to compare the primary models with freshly isolated tracheal tissue (respiratory mucosa) and with the permanent airway cancer cell lines RPMI 2650 and CaLu3. As we have demonstrated previously transcytosis of IgG antibodies through airway mucosa models, we additionally investigated the expression of FcRn and FCGRs [8, 21].

**Fig. 2:**
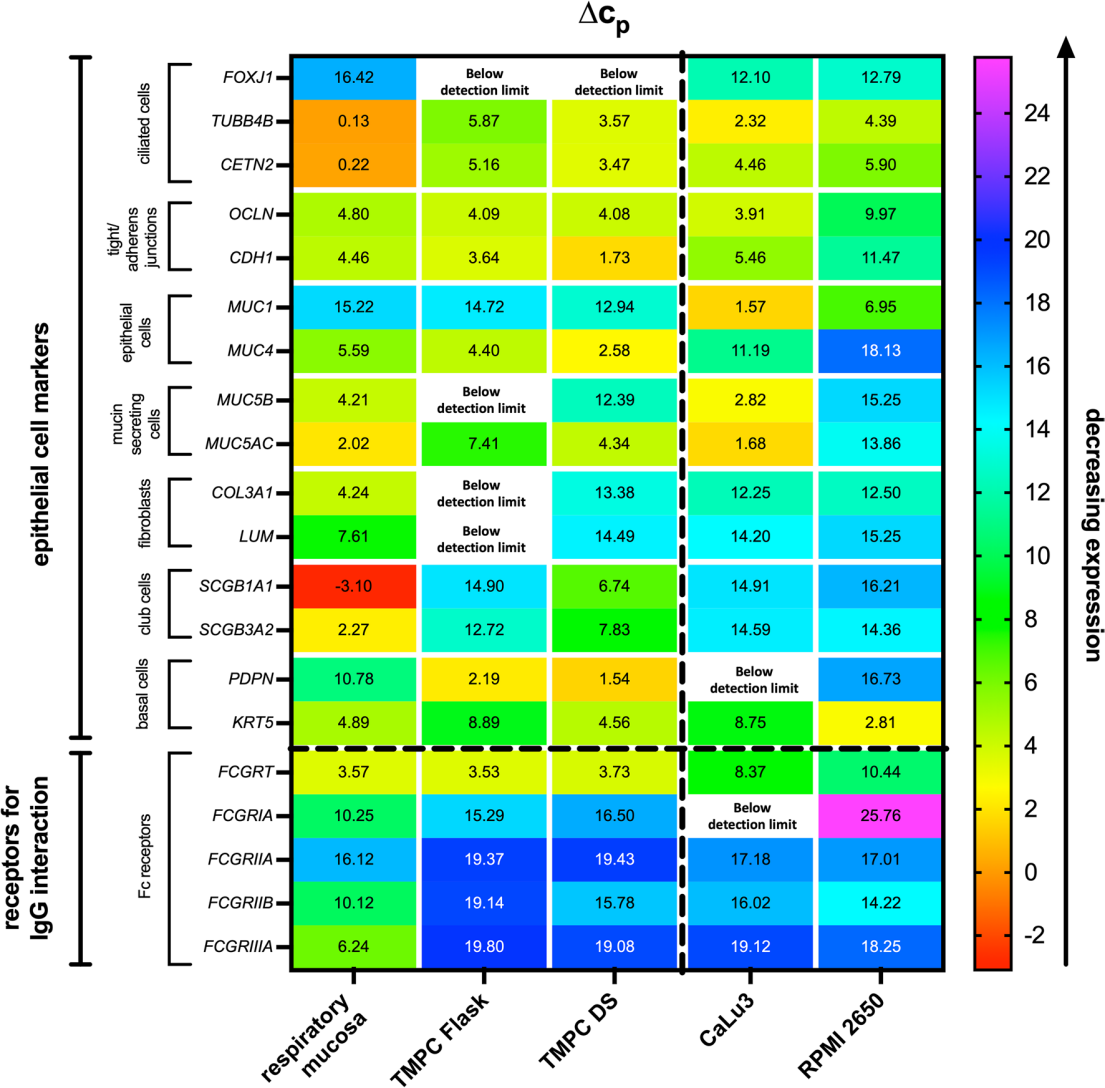
Expression analysis of epithelial markers by RT-qPCR of the different cellular models and respiratory mucosa from trachea for comparison. Expression levels were referenced to GAPDH as housekeeping gene and are expressed as ∆*c*_p_ with low values for a strong expression of the marker and high values for a weak expression of the indicated mRNA (see color scale on the right side). Relevant markers for different mucosal cell types as indicated on the left side were analyzed, but also expression of Fc receptors since they are relevant for IgG uptake, distribution, and elimination in airway mucosa. Values are shown as mean. Trachea tissue: *n* = 6–12, *N* = 2–4; TMPC Flask method: *n* = 9–12, *N* = 3–4; TMPC DS: *n* = 6–12, *N* = 2–4; RPMI 2650: *n* = 4–12, *N* = 1–3; CaLu3: *n* = 4–12, *N* = 1–3. Two-way ANOVA was performed to analyze the effects of two independent factors (cell type/cultivation method and gene marker) on a dependent variable simultaneously. Multiple comparison for the statistical analysis revealed a significant difference (****p ≤ 0.0001) between cellular models and cultivation methods

Figure 2 displays the results of the RT-qPCR as Δ*c*_p_ value [40]. The RT-qPCR analysis revealed significant differences between the various cellular models, particularly when compared to respiratory mucosal tissue.

A rather strong expression of the genes *TUBB4B* [41] and *CETN2* [42] (known to be involved in ciliogenesis) was observed in freshly isolated respiratory mucosa from the trachea. *FOXJ1* was only expressed at lower levels (Fig. 2) [43, 44]. These markers for ciliated cells showed reduced expression in both TMPC models compared to the tissue. *FOXJ1* expression was even below the detection limit indicating a low abundance of ciliogenesis, thus, the presence of ciliated cells in the primary models. Previous studies revealed *FOXJ1* to be expressed in primary cell culture as well [45]. Therefore, increasing the number of *FOXJ1*-expressing cells might represent a potential improvement for the TMPC models. RPMI 2650 and CaLu3 demonstrated significantly elevated expression of F*OXJ1* but also decreased expression of *TUBB4B* and *CETN2* compared to mucosal tissue. Expression of tubulin (gene product of *TUBB4B*) and Centrin 2 (gene product of *CETN2)* is described in ciliated tissue as these proteins are components of the cilia [46, 47]. Ledizet et al*.* also noted constant expression of Centrin 2 in human tracheal epithelial cells, with an increase observed at the onset of ciliogenesis. *FOXJ1*, a crucial transcription factor in ciliogenesis, is known to precede the actual process of ciliogenesis [44]. Δ*c*_p_ values of 16.42 ± 0.53 (mucosal tissue), 12.10 ± 2.13 (Calu3), 12.79 ± 0.90 (RPMI2650), and undetectable in the TMPC models indicate only medium to low expression of *FOXJ1*. This could explain the expression pattern in all cellular models investigated and in the tracheal tissue since transcription factors do not require a strong expression to induce the downstream-regulated gene [48]. Also, *FOXJ1* is not the sole influencer of ciliogenesis; other factors such as the microenvironment of the cells, including growth factors and the extracellular matrix, may also impact cilia formation [49, 50]. Likewise, serum starvation has been reported to induce ciliogenesis [51, 52], likely by arresting more cells in the G0 phase or due to the absence of secreted factors inhibiting ciliogenesis [53].

Tight and adherens junctions create intercellular contacts between the cells and thereby they are highly relevant for the formation of an epithelial barrier. Occludin encoded by *OCLN* is one of the most relevant tight junction forming proteins [54, 55]. Cadherin-1 encoded by *CDH1* is a cell–cell adhesion protein with a high turnover rate [56]. Since tight and adherens junctions are important factors for paracellular transport, their expression was analyzed [57, 58]. Comparable expression levels of *OCLN* around a Δ*c*_p_ of 4 were detected in tracheal tissue, both TMPC models, and CaLu3, whereas significantly far lower levels were observed in RPMI 2650 (****p ≤ 0.0001). *CDH1* expression was similar in the tissue, TMPC Flask, and CaLu3, but also significantly decreased in RPMI 2650 (****p ≤ 0.0001). Interestingly, TMPC DS displayed higher expression levels than its parental tissue (****p ≤ 0.0001 vs. tracheal tissue and **p ≤ 0.002 vs. TMPC Flask) indicative for tighter cell–cell connections in this model.

Respiratory mucins can be categorized into two major groups: cell-tethered mucins such as encoded by *MUC1* and *MUC4* (produced mainly by epithelial cells) [59] and secreted gel-forming as encoded by *MUC5B* and *MUC5AC* [60]. Membrane-anchored mucins such as MUC1 [61] and MUC4 [62] are indicative for epithelial cells. Compared to the respiratory mucosa from the trachea, comparable levels of both mucin mRNAs were detected in TMPC Flask, while slightly higher levels were observed in TMPC DS (*p ≤ 0.02 for *MUC1* and ***p ≤ 0.0005 for *MUC4*). The trachea and both TMPC models exhibited higher expression of *MUC4* than *MUC1*, consistent with previous studies indicating *MUC4*’s prevalence in larger conducting airways [63]. Despite this mild deviation in the DS model, strong changes in expression levels were found in the cancer cell models CaLu3 and RPMI 2650: significantly higher levels of *MUC1* (both ****p ≤ 0.0001 compared with respiratory mucosa) and significantly lower levels of *MUC4* (both ****p ≤ 0.0001 compared with respiratory mucosa from trachea). CaLu3 and RPMI 2650 expressed both more *MUC1* than *MUC4* as described previously [25]. Furthermore, it has been described that lung adenocarcinomas (origin of CaLu3) express more *MUC1* than squamous or adenosquamous carcinomas (origin of RPMI 2650) which fits well our observations [64].

The gel-forming secreted mucins are either produced in submucosal glands as Mucin 5B (MUC5B) or in goblet cells as Mucin 5AC (MUC5AC) [15, 65–67]. While high levels of mucin mRNAs were detected in tracheal tissue, both TMPC models showed significantly lower levels. *MUC5B* was even below the detection limit in TMPC Flask (****p ≤ 0.0001 compared with respiratory mucosa). Both MUC5AC and MUC5B have been reported to be expressed in human airway primary cells cultivated in a comparable manner [45]. Thus, increasing the number of bucket cells could be an interesting point of improvement for the TMPC models described here. CaLu3 has comparable expression levels as the tissue and RPMI 2650 significantly lower ones (****p ≤ 0.0001). Comparable levels of *MUC5AC* in the trachea were only detected in CaLu3 while the TMPC Flask was significantly reduced (****p ≤ 0.0001). TMPC DS showed a non-significant tendency for reduced levels and a dramatic reduction in RPMI 2650 (****p ≤ 0.0001). This is an indication, that only low numbers of mucus-secreting cells are part of the TMPC models, potentially due to the differentiation of basal cells leading more likely to goblet cell characteristics instead of glandular mucous cells. Since CaLu3 is a cell line derived from an adenocarcinoma of the bronchial submucosal glands, it is not surprising that the CaLu3 showed very high levels of the secreted mucins under ALI conditions [68]. Additionally, our findings are supported by previous studies [25, 69, 70]. Due to their origin, the RPMI 2650 showed the lowest levels of *MUC5AC* and *MUC5B*. However, the extent to which RPMI 2650 are able to produce the secreted mucins is controversially debated in literature [19, 25].

Fibroblasts are cells of the *lamina propria* that produce collagen and other components of the extracellular matrix [71, 72]. Since fibroblasts are very robust cells, they are able to overgrow primary cell culture. Hence, an analysis of *COL3A1* (Type III Collagen) and *LUM* (Lumican; involved in collagen fibril organization) gene expression is indicative for fibroblasts as both are exclusively synthetized by fibroblasts. All cellular models exhibited a significantly reduced expression of both, *COL3A1* and *LUM* (TMPC Flask even undetectable levels; ****p ≤ 0.0001), demonstrating that, if any, only traces of fibroblasts are detectable in the ALI model. This might be due to the density gradient centrifugation (steps 3a and 3b in Fig. 1) during purification [73]. The need for fibroblast removal in primary epithelial cell cultures is well documented [74, 75]. Therefore, the fibroblast removal method described here may also be a valuable approach for other studies. The low levels of fibroblast markers in CaLu3 and RPMI 2650 are not surprising, given their non-fibroblastic tumor origins [76–79].

Club cells are smaller exocrine cells with short microvilli found in the smaller conducting airways but also may have progenitor cell functions*.* These abilities enable club cells to repair damages in the respiratory epithelium and to suppress inflammation and obstruction in the airways [80]. Secretoglobin family 1A member 1 (encoded by *SCGB1A1*) is a marker for club cells [81]. Secretoglobin family 3A member 2 encoded by *SCGB3A2* is another airway club cell marker [82]. Both markers were strongly expressed in tracheal tissue with the lowest Δ*c*_p_ values of the whole analysis for *SCGB1A1*. All cellular models displayed significantly reduced levels of both markers (****p ≤ 0.0001), although the decrease was less pronounced in TMPC DS.

Podoplanin is a mucin-type protein encoded by *PDPN* in basal cells of several tissues such as the airways [83]. Keratin 5 (also known as Cytokeratin-5) encoded by *KRT5* is a component of the cytoskeleton of basal epithelial cells [82, 84]. While the trachea showed a lower expression of *PDPN,* with a Δ*c*_p_ of 10.78 ± 0.985, both TMPC models exhibited significantly higher compared to the parental tissue (****p ≤ 0.0001). This is likely due to the survival of mainly basal cells during the culture, which then differentiate as progenitors into other cell types [15, 61, 62]. In contrast, both cancer cell models revealed a low expression of *PDPN* (****p ≤ 0.0001) and even undetectable expression in CaLu3. Remarkably, expression levels of the second basal cell marker *KRT5* were comparable in TMPC DS. TMPC Flask and CaLu3 showed slightly higher Δ*c*_p_ values (****p ≤ 0.0001). Lower Δ*c*_p_ values were found in RPMI 2650 (*p ≤ 0.02 for RPMI 2650). These findings match previous studies [76, 77, 85–87]. A somewhat similar expression pattern was observed for the mucin-producing cell markers as for *KRT5* [88]. It has been proposed that *KRT5* + basal cells act as progenitor cells for club cells. Furthermore, during differentiation, the expression of both *KRT5* and *SCGB1A1* was reported [89]. This could explain the different expression patterns of *KRT5* and *PDPN* and that *KRT5* may not be a specific basal cell marker [76, 77, 86, 87].

Finally, expression of Fc receptors, which is crucial for the uptake, transport, and elimination of IgG antibodies in the airways, was analyzed. FcRn encoded by *FCGRT* was previously shown as a highly relevant transporter of IgG to be expressed in endothelial cells, glands, and epithelial cells [21, 67, 90, 91]. *FCGRT* expression levels were comparably high in the trachea and both TMPC models. Significantly lower expression was observed in the permanent cell lines (****p ≤ 0.0001 vs. tissue) making the TMPC models more suitable for transmucosal IgG drug delivery studies. Furthermore, the respiratory mucosa contains various Fc gamma receptors (FCGRs, encoded by *FCGRIA, FCGRIIA, FCGRIIB*, and *FCGRIIIA*) likely to be expressed on mucosal immune cells for immune surveillance. These FCGRs can bind and internalize immune complexes but, in the case of FCGRI, have also been described to bind monomeric IgG molecules [92]. Not only immune cells express FCGR but also epithelial cells express some FCGRs, in particular FCGRII [23, 93]. Respiratory mucosa from the trachea showed higher expression of *FCGRIIIA*, modest levels of *FCGRIA* and *FCGRIIB*, and rather low levels of *FCGRIIA*. As expected for all epithelial models without *lamina propria* and lymphoid follicles, expression levels of all FCGR decreased significantly (****p ≤ 0.0001 vs. tissue), except for comparable levels of *FCGRIIA* in CaLu3 and RPMI 2650. It is likely that immune cells from the tissue do not fully survive ALI conditions, although co-cultivation of antigen-presenting cells at ALI has been described [94]. FCGRIa showed the highest remaining expression in both TMPC models. Therefore, FCGRIa might be an interesting candidate to be investigated for its effect on IgG transport through the respiratory epithelium. FCGRIIb has been shown to be responsible for IgG transport through the placenta [23] and has been discussed to play a role in the nasal mucosa [8].The FCGRIIb expression levels differed between TMPC Flask and DS model (***p ≤ 0.001). In general, the expression of FCGRs in non-myeloid cells is yet not well investigated. Nevertheless, it has been reported that keratinocytes, astrocytes, liver cells, sensory neurons, endothelial cell, and salivary gland epithelial cells express FCGRs as well [22]. As the expression levels of secretory cells and fibroblasts were higher in the DS method, it appears reasonable that these cells also displayed higher expression levels of FCGRIIb. Understanding the distribution and function of the different Fc receptors in IgG uptake in the respiratory mucosa is essential in order to develop anti-infective products for the prevention/therapy of infectious respiratory diseases [95]. Further, it is important for the biotechnological tailoring of IgG products intended for respiratory drug delivery [8]. With the binding to FcRn, IgG transcytosis is enabled in both directions across the polarized epithelium [95]. This enables endogenous/parenteral applied IgG to be transported to the mucosal surface to elicit protection. Further FcRn binding has been shown to facilitate the transport of immune complexes to immune-competent cells and can inhibit the intracellular replication of viruses [95–99]. FcRn is also expressed on myeloid-derived hematopoietic cells like monocytes, macrophages, and subsets of dendritic cells [100, 101]. With these cells, just like in endothelial and epithelial cells, FcRn is responsible for recycling and salvaging monomeric IgG but can also enhance the uptake of immune complexes and therefore antigen presentation and T cell activation [98, 102–104]. Nevertheless, it should be noticed that FcRn may be harmful in the form of so-called antibody-dependent enhancement (ADE) [105]. Thus, antibody engineering to increase FcRn binding has been shown to extend IgG half-life and mucosal uptake. Further, it may reduce the necessary dose but must be closely evaluated for safety and efficacy in case of anti-infectious antibodies [95]. FcRn can also be exploited in the form of Fc-fusion proteins and/or local vaccination strategies [106–109]. We have previously pointed out that the distribution of FCGRs within different cell and tissue types is not yet fully understood and focus of research [8, 22]. Additionally, genetic variations and, in immune cells, different stages of activation have been reported to attribute to the complexity of FCGRs’ expression patterns [110–112]. In terms of respiratory drug delivery, binding of IgG to FCGRs might have to be regarded ambivalently, depending on the therapy’s indication. With anti-infective therapies, FCGRs are essential for the uptake of immune complexes, antigen presentation, and further induction of immune responses (Fc effector functions) [110, 113]. In the treatment of other diseases, like cancer or the nose-to-brain approaches, FCGRs can contribute to the immunological effectiveness against, e.g., tumor cells. On the other hand, FCGRs might also result in increased degradation of the monomeric IgG [21]. For instance, in a previous study, we have observed that so-called lymphoid follicles were spared from IgG immunoreactivity after IgG uptake in porcine ex vivo nasal tissue, while IgG was well detectable in the *surrounding lamina propria* potentially attributed to IgG degradation in immune cells [21].

### Morphological characterization of specific airway epithelia markers

**Fig. 3:**
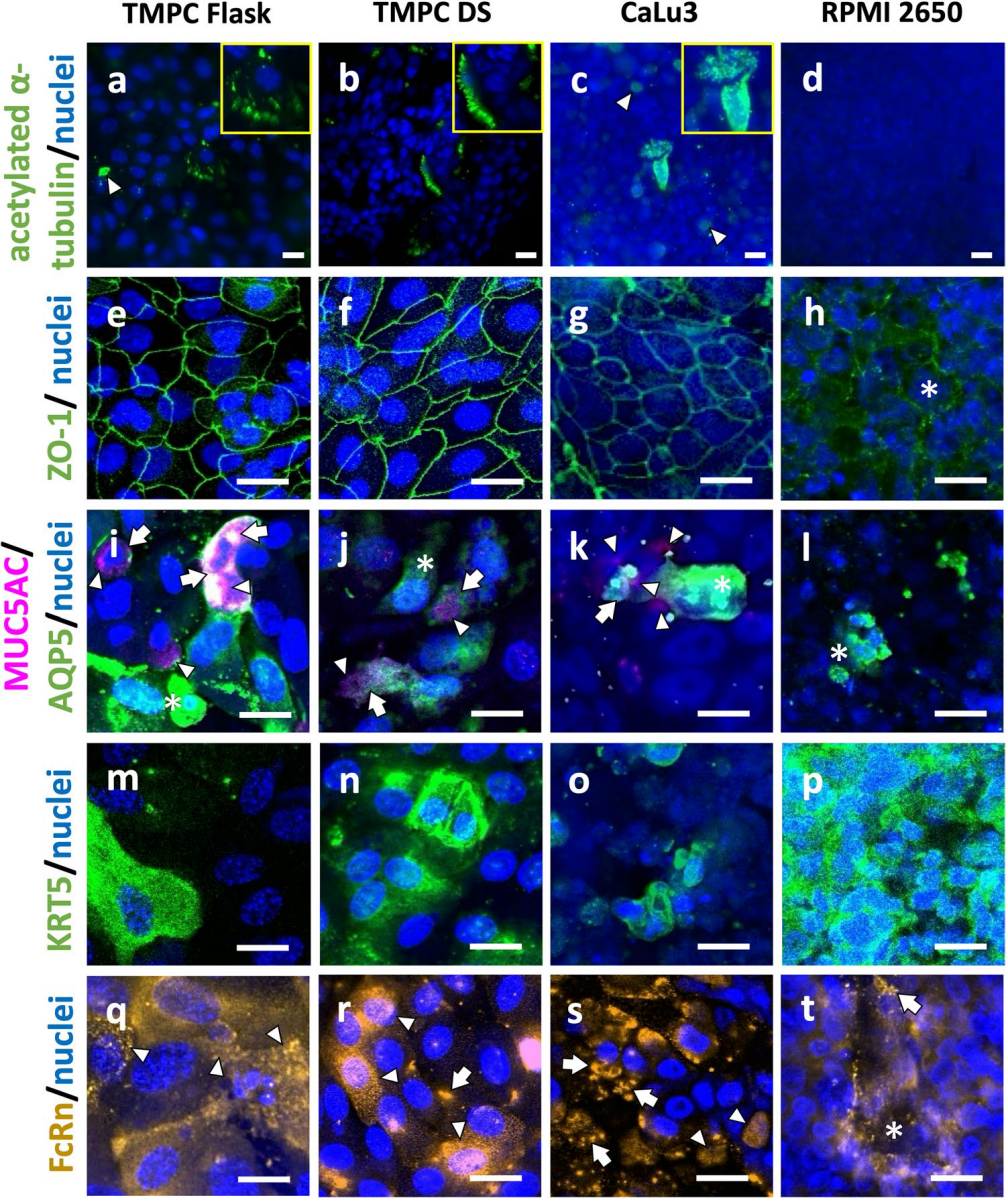
Representative images of immunoreactivities against several characteristics of epithelial cells with nuclei counterstained with DAPI in blue: **a**–**d** acetylated tubulin as component of cilia is organized in hairy-like structures (zoom-in in yellow boxes) as observed in up to 5% of TMPC Flask (**a**), TMPC DS (**b**), and in some CaLu3 cells (**c**), but also non-hairy like immunoreactivity (arrowheads) was observed in some TMPC Flask (**a**) and in many CaLu3 cells (**c**). No immunoreactivity at all was detected in RPMI 2650 (**d**). **a**–**d** Taken using a 40-fold air objective. **e**–**h** ZO-1 indicative for the formation of tight junctions was observed in the typical cobblestone pattern in both TMPC models (**e**, **f**) and CaLu3 (**g**), but only a few RPMI 2650 cells were found that were surrounded by a diffuse ZO-1 immunoreactivity (asterisks in **h**). Due to their growth in multiple layers, the images of CaLu3 and RPMI 2650 appear blurrier as many cells are above or beneath the focus. In contrast, the monolayers of the primary TMPC models produce sharp images. **e**–**h** Taken using a 20-fold air objective. Mucus-secreting cells were identified by immunoreactivity against the mucin MUC5AC (arrowheads pointing to pink structures in **i**–**k**) as well as against the water channel aquaporin-5 (AQP5; asterisks in **i**–**l**). While RPMI 2650 predominantly showed immunoreactivity against AQP5 (asterisks in **l**), double-positive cells were observed in both TMPC models and CaLu3 (see arrows in **i**–**k**). **m**–**p** Cytokeratin 5 (KRT5) as marker for basal cells was detected in some cells of both TMPC models and in CaLu3 and in all investigated RPMI 2650 cells. **m**–**p** Taken using a 20-fold air objective. **q**–**t** Uptake of IgG was recently demonstrated to be mediated in the airway mucosa via FcRn, and comparable with the qPCR data all four models showed a robust immunoreactivity against FcRn. However, only the both TMPC models demonstrated an endosomal-like staining pattern (arrowheads in **q**–**s**), while a more cluster-like staining pattern was found in CaLu3, TMPC Flask, and RPMI 2650 (arrows in **r**–**t**), and a diffuse staining was observed in RPMI 2650 (asterisks in *t*). **q**–**t** Taken using a 20-fold air objective. All scale bars: 20 µm

In order to visualize and confirm the results obtained via RT-qPCR, immunoreactivity against different epithelial markers was performed with all cellular models grown on membrane inserts and differentiated under ALI conditions. Based on the RT-qPCR data for ciliated cells, the cell models were stained for acetylated tubulin as cilia component. In contrast to abundantly ciliated tissue from the conducting airways (see Supplementary information 2a), only a few hair-like structures with immunoreactivity against acetylated tubulin were observed in TMPC DS and TMPC Flask, presenting about 5% of all cells (see arrows in Fig. 3a, b). The cilia staining itself can be compared to previously published data in sheep and human primary cells, although the amount of ciliated cells is reduced in the models described here [114, 115]. During cultivation in flasks, beating cilia can be observed by light microscopy as described previously for undifferentiated nasal primary epithelial cells [19]. The presence of non-beating cilia is hard to evaluate in membrane inserts since the pores of the membrane inserts interfere with a proper light microscopic inspection of such thin structures. However, beating cilia are easier to identify by light microscopy of membrane inserts by their constant movement. A few days after seeding the flask-cultivated cells in membrane inserts (TMPC Flask model), such beating cilia can be observed but are lost after the first week of ALI differentiation.

Interestingly, as reflected by their higher expression levels of tubulin by RT-qPCR (Fig. 2), even more immunoreactivity against acetylated tubulin was observed in CaLu3; however, this was predominantly a cytosol staining (arrowhead in Fig. 3c) and rarely associated with a hairy like morphology (zoom-in in yellow box in Fig. 3c). This implies that the presence of tubulin is not automatically leading the formation of cilia. Also, during maintenance of the CaLu3 cells in flasks, no visible signs of beating cilia could be observed by light microscopy. In TMPC, detection of acetylated tubulin in soma was only detectable in very few cells (zoom-in in yellow box in Fig. 3a and b).

No traces of acetylated tubulin were detected in ALI-differentiated cultures of RPMI 2650 (Fig. 3d) nor any signs for cilia during maintenance of undifferentiated cells in flasks.

Together with mucus, cilia are responsible for mucociliary clearance (MCC) in the airways. MCC is the process responsible for the transportation of particles and pathogens towards the pharynx where they can be swallowed or coughed out [116]. Given the importance of cilia, their presence in an in vitro model would improve this model. Cozens et al*.* and O’Boyle et al*.* were able to detect about 60% of ciliated cells in a 21-day ALI cultivation of bovine and ovine airway primary cells [115, 117]. Different values for the in vivo cilia coverage of the airways ranging from 45% up to 90% can be found in previous publications [118–123]. Thus, improving the number of beating cilia represents an important parameter to improve the here described primary cell models.

The formation of tight junction was determined by immunoreactivity against *zonula occludens* protein-1 (ZO-1) [124]. For both TMPC models and the CaLu3 cells, a well-developed, net-like structure was observed for ZO-1 immunoreactivity in all cells investigated (see Fig. 3e–g). This characteristic structure was described previously in various studies [125–128]. For RPMI 2650, a more diffuse staining at the cellular borders was visible and could not be confirmed for all cells (see asterisk in Fig. 3h). These findings are in concordance with the expression data determined by RT-qPCR (Fig. 2). Tight junctions are part of the so-called junctional complex, which connects neighboring epithelial cells and forms a barrier and regulates paracellular transport [129]. Therefore, the development of tight junctions has a major impact on the barrier integrity of epithelia in vivo (see Supplementary information 2b) and in vitro. In addition, barrier integrity has an influence on the electrical resistance and thus on the permeability, especially in vitro.

Aquaporin 5 (AQP5) and MUC5AC are markers for mucin-secreting or goblet cells. MUC5AC immunoreactivity was detectable in both TMPC models and CaLu3 (see arrowheads in Fig. 3i–l), while hardly any MUC5AC-positive cells were detected in RPMI 2650. Again, this corresponds well to the results obtained via RT-qPCR, where the ∆*c*_p_ value for *MUC5AC* was significantly higher for RPMI 2650 compared to TMPC and CaLu3. Yonker et al*.* showed MUC5AC staining for a primary human airway model, revealing a pattern similar to the here described TMPC model [130]. Also, AQP5 immunoreactivity was observed in all four cellular models (exemplary structures marked by asterisk in Fig. 3i–l) comparable to earlier published data for mice primary epithelial cells [131]. MUC5AC and AQP5 staining was also performed in tracheal tissue revealing comparable structures (see Supplementary information 2c). AQP5 is one of many water channel proteins that has been described to be localized in cells of the epithelium of the conducting airways and alveolar region as well as in glands in the *lamina propria* [132]. It is described to be found in the larger airways to counterwork water loss and plays a part in the production and secretion of *MUC5AC* and *MUC5B* in the lungs [133]. However, co-localization of MUC5AC and AQP5 in subcellular structures was only observed in the TMPC Flask model, and both TMPC models showed double positive cells, however, with a different subcellular distribution of both markers (see arrows in Fig. 3i–j). Double-positive cells were a rather rare event in CaLu3 (Fig. 3k). Mucus and cilia are the key players involved in MCC; hence, the presence of mucus-producing goblet cells is important for an in vitro airway model [116]. Further, the secreted mucus has the ability to bind H^+^-ions to protect the tissue from pH shifts and increase viscosity [134]. Mucus production was previously observed in primary trachea cell culture from rats as well [135]. RPMI 2650 and CaLu3 are also reported to show mucus production [19].

Immunoreactivity against KRT5 as a marker for basal cells revealed a very strong staining pattern in PRMI 2650 (Fig. 3p) and a rather strong in the TMPC DS model (Fig. 3n). Interestingly, when TMPC were cultivated first in flasks before differentiation in ALI, they showed a significantly lower number of KRT5-immunoreactive cells (Fig. 3m). CaLu3, in addition, also displayed a similar lower number of KRT5-immunoreactive cells (Fig. 3o). IF staining of tracheal tissue showed KRT5 to be mainly located in the basal cell layer in the epithelium (see Supplementary information 2d). In summary, the morphological characterization aligns well with the RT-qPCR results (Fig. 2) and is consistent with previous studies illustrating KRT5 IF staining in nasal epithelial cells [136]. The expression of *KRT5* in CaLu3 is described differently depending on the literature. On one side, Lodes et al. described CaLu3 to be *KRT5*-negative. Our findings demonstrate a low immunoreactivity for KRT5 in differentiated CaLu3. On the other side, Effah et al*.* describe a decreasing *KRT5* expression over time [137].

Last but not least, immunoreactivity against FcRn (gene product of *FCGRT*) was determined as FcRn is described as a highly relevant IgG transporter in mucosa and epithelia cells [8, 21, 91, 138–141]. FcRn is reported to be distributed predominantly in endosomes and at the apical side in respiratory and olfactory mucosa cells (see Supplementary information 2e) [67]. Both TMPC models showed, as expected, an endosomal immunoreactivity pattern for FcRn (exemplary structures marked by arrowheads in Fig. 3q, r). This pattern was also observed for CaLu3 for certain extent and was described by Bequignon et al*.* previously for nasal epithelial cells [142]. However, in CaLu3, also larger clump-like subcellular structures were found (see arrows in Fig. 3s). Such clump-like structures were only occasionally detected in TMPC DS and absent in TMPC Flask (see arrow in Fig. 3r). In RPMI 2650, the staining pattern was highly diffuse (see asterisk in Fig. 3t), although an endosomal distribution appeared to be evident underneath with some clump-like structures (see arrow in Fig. 3t). While nearly all TMPC cells demonstrated a clear FcRn immunoreactivity, not all CaLu3 and RPMI 2650 cells displayed FcRn immunoreactivity.

### Characterization of barrier integrity of the epithelial airway models

**Fig. 4:**
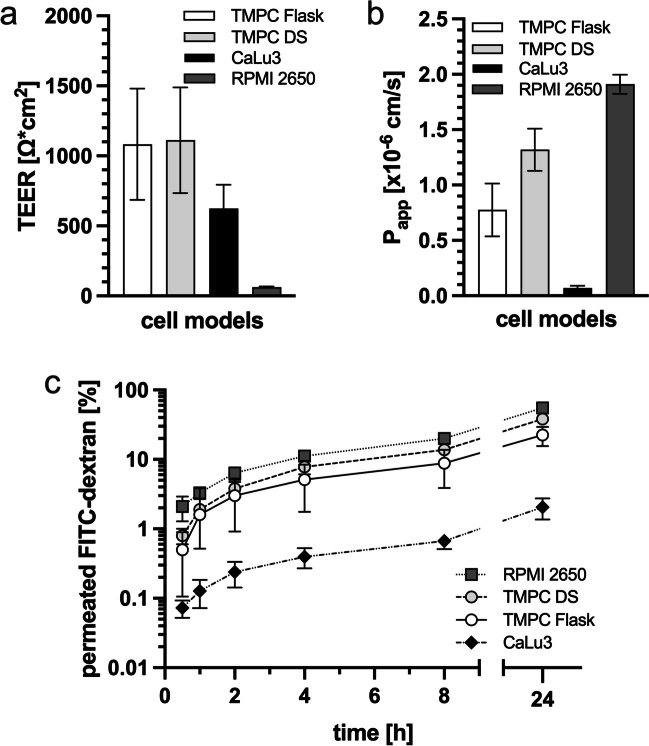
Characterization of electrical barrier integrity (TEER) and paracellular permeability. **a** TEER values of the investigated cell models were determined after 21 days of differentiation under ALI. Both primary models demonstrate very high TEER; CaLu3 show medium TEER values and RPMI 2650 very low values. It should be noted that CaLu3 and RPMI 2650 grow in multilayers presumably due to their cancer origin while the primary cells grow as monolayer; this may affect electrical resistance. All TEER values are shown as mean ± SD. Statistical significance is described in the text. TMPC Flask: *n* = 23, *N* = 1; TMPC DS: *n* = 69, *N* = 3; RPMI 2650: *n* = 30, *N* = 2; CaLu3: *n* = 60, *N* = 3. Ordinary one-way ANOVA was performed to evaluate potential significant differences between the different cellular models and cultivation methods. Multiple comparison for the statistical analysis revealed a significant difference in TEER (****p ≤ 0.0001) between all cellular models and cultivation methods. **b** Paracellular permeability of the 4 kDa tracer FITC-dextran through the different cellular models from the apical to the basolateral compartment. CaLu3 demonstrated here the tightest paracellular barrier, followed by TMPC Flask, TMPC DS, and finally RPMI 2650 being most leaky for the polar FITC-dextran. All values are mean ± SD; TMPC Flask/TMPC DS: *n* = 23, *N* = 1; CaLu3: *n* = 73, *N* = 3; RPMI 2650: *n* = 30, *N* = 1. Ordinary one-way ANOVA was performed to evaluate potential significant differences between the different cellular models and cultivation methods. Multiple comparison for the statistical analysis revealed a significant difference in *P*_app_ (****p ≤ 0.0001) between all cellular models and cultivation methods. **c** Kinetics of FITC-dextran permeability over 24 h expressed as percentage permeated from apical to basolateral demonstrate parallel kinetics in all models. Values are shown as mean ± SD. Both TMPC DS and TMPC Flask; CaLu3: *n* = 36, *N* = 2, *n* = 23, *N* = 1; RPMI 2650: *n* = 30, *N* = 1. Two-way ANOVA was performed to analyze the effects of two independent factors on a dependent variable (cell type and time) simultaneously. Multiple comparison for the statistical analysis revealed a significant difference in permeability (****p ≤ 0.0001) between all cellular models and cultivation methods

Barrier integrity is an important parameter for epithelial models since the epithelial border must prevent the invasion of pathogens or leakage of liquids [143]. One characteristic of cells grown under ALI conditions is the transepithelial electrical resistance (TEER). According to Srinivasan et al., the TEER value corresponds to the integrity of tight junctions connecting the epithelial layer; thus, TEER measurement is a routine quality control to evaluate the electrical barrier function in cell culture models [144]. When comparing TEER of the two different cultivation methods of the tracheal primary model TMPC Flask and TMPC DS after 21-day differentiation under ALI, both methods achieved comparable values (Flask: 1083 ± 397 Ω*cm^2^; DS 1112 ± 378 Ω*cm^2^; n = 23–69; Fig. 4a), while both permeant cell lines resulted in significant lower levels (CaLu3: 624 ± 170 Ω*cm^2^; RPMI 2650: 58 ± 5 Ω*cm^2^; n = 30–60; Fig. 4). The TEER values of both TMPC Flask and TMPC DS are within the range of 700–1200 Ω*cm^2^ described for human trachea primary cells after 14-day ALI by Pezzulo et al. [122]. These values are somewhat higher than our previously reported nasal primary models from respiratory mucosa (846 ± 550 Ω*cm^2^) and olfactory mucosa (648 ± 371 Ω*cm^2^), indicating the tendency that respiratory epithelium forms tighter electrical barriers [19]. It should be noted that after 22 days of ALI differentiation, the TEER dropped under the threshold of 500 Ω*cm^2^ (data not shown); thus, TMPC as described here should not be used after 21 days.

TEER data of CaLu3 obtained in this study are within the published range, but reported TEER values vary considerably from study to study [145]. Interestingly, TEER values of the TMPC models were significantly higher than from CaLu3, even though the TMPC grow primarily in monolayers whereas CaLu3, derived from a bronchial adenocarcinoma, displayed multilayers after 21 days as demonstrated by a z-stack video (Supplementary information 3). CaLu3 are described to form polarized cell layers with many characteristics of respiratory epithelium when cultivated under ALI conditions [15]. They form tight junctions, produce mucins, and in some cases, even cilia-like structures were observed [145]. TEER values of RPMI 2650 were significantly lower than any other tested cellular model and comparable to a previous study. Although RPMI 2650 are derived from an anaplastic squamous cell carcinoma of the human nasal septum, they are used as a common model for nasal respiratory cells [11, 12]. Interestingly, when RPMI 2650 cells were seeded with a higher passage of 54, the observed TEER levels dropped even lower (26 ± 4 Ω*cm^2^) than at the usual passage below 18 [10, 124]. A drop of TEER is usually reported with higher passage numbers [144]. Nevertheless, also higher TEER values are reported, which are associated with morphological changes in electron microscopy in Caco-2 (human colon adenocarcinoma cell line) [146]. Interestingly, the expression data of the tight/adherens junction markers *OCLN and CDH1* (Fig. 2) reflect well the TEER measurements: significant differences in *OCLN* expression were only observed compared to RPMI 2650, which had the lowest TEER value and highest ∆*c*_p_ values. For *CDH1*, TMPC DS exhibited significantly higher expression, while RPMI 2650 showed significantly lower expression. These results suggest that the barrier of the TMPC models and CaLu3 is comparable to tissue. However, the adherence among cells for signaling, among other functions, may differ, despite both types of cell–cell connections originating from the same precursor junction [147]. ZO-1 immunoreactivity confirmed these findings, with both TMPC models and CaLu3 exhibiting characteristic net-like staining for tight junctions, while RPMI 2650 displayed a more diffuse staining pattern. When comparing the nasal mucosa’s barrier function to other mucosal parts, such as different intestinal sections, nasal tissue demonstrated the lowest electrical resistance [148], indicating that RPMI 2650 resemble its leaky origin [149].

To further characterize the barrier formation of the different models, permeability of FITC-dextran with a molecular weight of approximately 4 kDa was assessed as reporter for paracellular transport [150]. The *P*_app_ value describes the flux through a barrier [125]. *P*_app_ values presented in Fig. 4b were calculated based on the permeability kinetics shown in Fig. 4c. Briefly, the TMPC DS model demonstrated a significantly higher *P*_app_ value (TMPC Flask: 0.775 ± 0.2386 cm/s; TMPC DS: 1.319 ± 0.1904 cm/s; *n* = 19–23; Fig. 4b), thus higher permeability than the Flask cultivation (TMPC Flask: 22.5 ± 6.9%; TMPC DS: 38.3 ± 5.5%, *n* = 23; ****p ≤ 0.0001; Fig. 4c). Although CaLu3 do not form a very high electrical barrier (Fig. 4a), they had the lowest *P*_app_ value (0.070 ± 0.0218; *n* = 73; ****p ≤ 0.0001 compared to all other models; Fig. 4b) and lowest observed permeability after 24 h with 2.05 ± 0.69% (*n* = 73; ****p ≤ 0.0001 compared to all other models; Fig. 4c) 4 kDa FITC-dextran analyzed from the basolateral/luminal compartment. In a study of Grainger et al., CaLu3 cell line cultured for 11 days under ALI was used and described a *P*_app_ of ~ 10^−7^ cm/s for tracers with a molecular weight of ~ 4 kDa somewhat higher than observed in the present study (~ 0.7 × 10^−7^ cm/s) [145]. The differences may be due to the longer cultivation time, which may result in multilayered cell culture for CaLu3 (Supplementary information 3).

Last but not least, more than 50% of the FITC-dextran molecules permeated from the luminal/apical compartment through the RPMI 2650 layer to the basolateral (55.5 ± 2.8%; Fig. 4c) within the same time resulting in a *P*_app_ of 1.911 × 10^−6^ ± 0.0867 × 10^−6^ cm/s (*n* = 30; ****p ≤ 0.0001 compared to all other models; Fig. 4b). A passage number of 54 in RPMI 2650 demonstrated hardly any barrier anymore with a FITC-dextran permeability of 98.9 ± 6.9% (*n* = 23). Data from previous studies showed that *P*_app_ depends on the molecular weight of the applied molecule and on the cell line/type used for the experiment [151–154]. For RPMI 2650 cells, *P*_app_ values between 5.07 × 10^−6^ ± 0.01 × 10^−6^ cm/s and 16.1 × 10^−6^ ± 0.1 × 10^−6^ cm/s are reported [155]. Therefore, the here observed value of 7.14 × 10^−6^ ± 0.34 × 10^−6^ cm/s for RPMI 2650 is located within this range. Based on a study of Sibinovska and co-workers, the *P*_app_ value indicates the degree of paracellular drug permeability with high (~ > 0.75 × 10^−6^ cm/s), moderate (~ 0.75 × 10^−6^ cm/s > *x* > 0.25 × 10^−6^ cm/s), and low (~ < 0.25 × 10^−6^ cm/s) permeability [154]. According to this classification, both TMPC models and RPMI 2650 cells showed a high permeability while CaLu3 demonstrated a moderate permeability for FITC-dextran.

When correlating the initial TEER values after 21 days under ALI conditions with the paracellular FITC-dextran permeability after 24 h, weak but significant negative correlations were observed for TMPC Flask (**p ≤ 0.003; *r*^2^ = 0.3631; *n* = 23; Supplementary information 4a) and TMPC DS (**p = 0.004; *r*^2^ = 0.4337; *n* = 17; Supplementary information 4a), indicating that a tighter electrical barrier correlates significantly with a tight paracellular barrier integrity in these primary cell models. A similar negative correlation was observed in a previous study with nasal primary airway cells [19]. Further, our data demonstrate that with an initial TEER value lower than ~ 500 Ω*cm^2^, the chance of highly increased FITC-dextran permeability was more likely; thus, lower TEER than 500 Ω*cm^2^ may be indicative for a leaky barrier. Moreover, TEER values higher than 500 Ω*cm^2^ resulted in less variability of permeated FITC-dextran. Therefore, we recommend that both TMPC Flask and TMPC DS should not be used for assays or experiments, if the initial TEER value is below 500 Ω*cm^2^ after 21 days under ALI differentiation. No significant correlation (*p* = 0.17; *r*^2^ = 0.05; *n* = 40; Supplementary information 4b) was observed for CaLu3 as expected from the significant lower initial TEER values and the lower amount of permeated FITC-dextran. Also, initial TEER of RPMI 2650 did not correlate with permeability (*p* = 0.18; *r*^2^ = 0.06; *n* = 29; Supplementary information 4b).

For small molecules, the barrier integrity is a major cellular factor influencing permeability [156]. Since the TEER measurements of the TMPC Flask and TMPC DS were similar, it could be suggested that similar amounts of FITC-dextran would permeate in both methods. But the experimental data demonstrated that despite both TMPC models displayed a correlation between initial TEER and permeability, the percentage of permeability differed significantly from 4 to 24 h. Hence, it should be considered that apart from TEER, other parameters like cell size, tight junction length during differentiation, transcellular transport, and expression of influx/efflux transporters may also impact permeability [157–161]. Furthermore, it was recently demonstrated that the permeation behavior of different so-called paracellular tracers may diverge in different cell types [160]. Interestingly, we also have unpublished observation that medium may significantly impact the outcome of TEER and permeability experiments, so this data should be only compared within the same culture and medium conditions.

Similar as for TEER, also FITC-dextran permeability in RPMI 2650 displayed a strong dependence on the passage number with 55.5 ± 3.0% of permeated FITC-dextran within 24 h and 98.9 ± 6.9% for passage 54 under the same conditions.

## Conclusion

To give the most accurate predictions for later in vivo studies, in vitro models should resemble the tissue of interest as closely as possible. In this study, we analyzed and characterized two differently cultivated TMPC models and two permanent airway cell lines and compared them to the original tissue of the porcine trachea.

This study aimed to establish and characterize an in vitro model for TMPC cultivation based on the existing model described by Ladel et al*.* [19]. An adapted protocol for TMPC’s isolation, purification, and cultivation was successfully and reproducibly established.

RT-qPCR and immunoreactivity against airway epithelial marker revealed significant differences between the primary TMPC models and the established cell models CaLu3 and RPMI 2650 with primary cells reflecting the airway-specific markers and receptors for therapeutic IgG uptake far better than the established cell lines. Some smaller differences between the two TMPC cultivation methods were observed, indicating an influence of the flask cultivation step on the cell differentiation.

Further studies and research are needed to improve the number of ciliated cells in this setup. Additionally, it will be of great interest to investigate the potential distribution of Fc receptors in the respiratory epithelium models and their contribution to the uptake, transcytosis, or degradation of applied IgG.

In summary, our findings highlight the superior performance of primary cell models in mimicking the in vivo conditions of respiratory airway mucosa, thus enhancing the translational potential of in vitro results to clinical settings. Key features such as the formation of a tight epithelial barrier, mucin production, and the presence of club/basal cells were better retained in primary cell models compared to permanent cell lines. Moreover, the expression of FcRn in primary cell models closely resembles that of respiratory mucosal tissue. This is a novel finding and crucial consideration when developing antibodies and antibody-based drugs for respiratory applications. In particular, the TMPC DS cultivation method represents a reliable, suitable, and time-efficient in vitro model.

## Supplementary Information

Below is the link to the electronic supplementary material.

Supplementary file1 Morphology of TMPCs after 8 h in culture flask, 40 × magnification, scale bar: 100 µm. (PNG 10703 KB)

Supplementary file2 Representative images of immunoreactivities against several characteristics of epithelial cells with nuclei counterstained with DAPI in blue for tracheal tissue: (a) acetylated tubulin as component of cilia is organized in hairy like structures. (b) ZO-1 indicative for the formation of tight junctions was observed in the typical apical location in the epithelium. (c) Mucus-secreting cells were identified by immunoreactivity against the mucin MUC5AC as well as against the water channel aquaporin-5 (AQP5). (d) Cytokeratin 5 (KRT5) as marker for basal cells was detected mainly in the basal cell layer of the epithelium. (e) Uptake of IgG was recently demonstrated to be mediated in the airway mucosa via FcRn, and comparable with the qPCR data, the epithelium showed a robust immunoreactivity against FcRn. All images were taken using a 40-fold air objective. All scale bars: 20 µm. (PNG 5843 KB)

Supplementary file3 Video of z-stack scan of ZO-1 (tight junctions) immunoreactivity in CaLu3, ZO-1 in green, nuclei are counterstained in blue with DAPI; 20 × magnification; scale bar: 100 µm. (MP4 9550 KB)

Supplementary file4 Correlation of FITC – dextran permeation after 24 h with initial TEER values for different cell culture models after 21 days differentiation under ALI cultivation. (A) A significant correlation was observed for TMPC DS and TMPC Flask while (B) no correlation was found for CaLu3 and RPMI 2650. TMPC DS: n = 23, N = 1; TMPC Flask, n = 23, N = 1. B RPMI 2650: n = 30, N = 1; CaLu3: n = 36, N = 2; ns, not significant. (PNG 1147 KB)

## Data Availability

The data can be obtained via the figshare.com repository with the following currently private links: https://figshare.com/s/456c63bc72cf73968ac9, https://figshare.com/s/e9d155813a22144d87d0, https://figshare.com/s/d64d28b3030df2936bde, https://figshare.com/s/4bf6e32874ae94f17d3b.
