# Supplementary figures and images for: Characterization of a primary cellular airway model for inhalative drug delivery in comparison with the established permanent cell lines CaLu3 and RPMI 2650
Source: In Vitro Model. 2024 Nov 25;3(4-6):183–203. doi: 10.1007/s44164-024-00079-y (PMC11756470; doi:10.1007/s44164-024-00079-y)

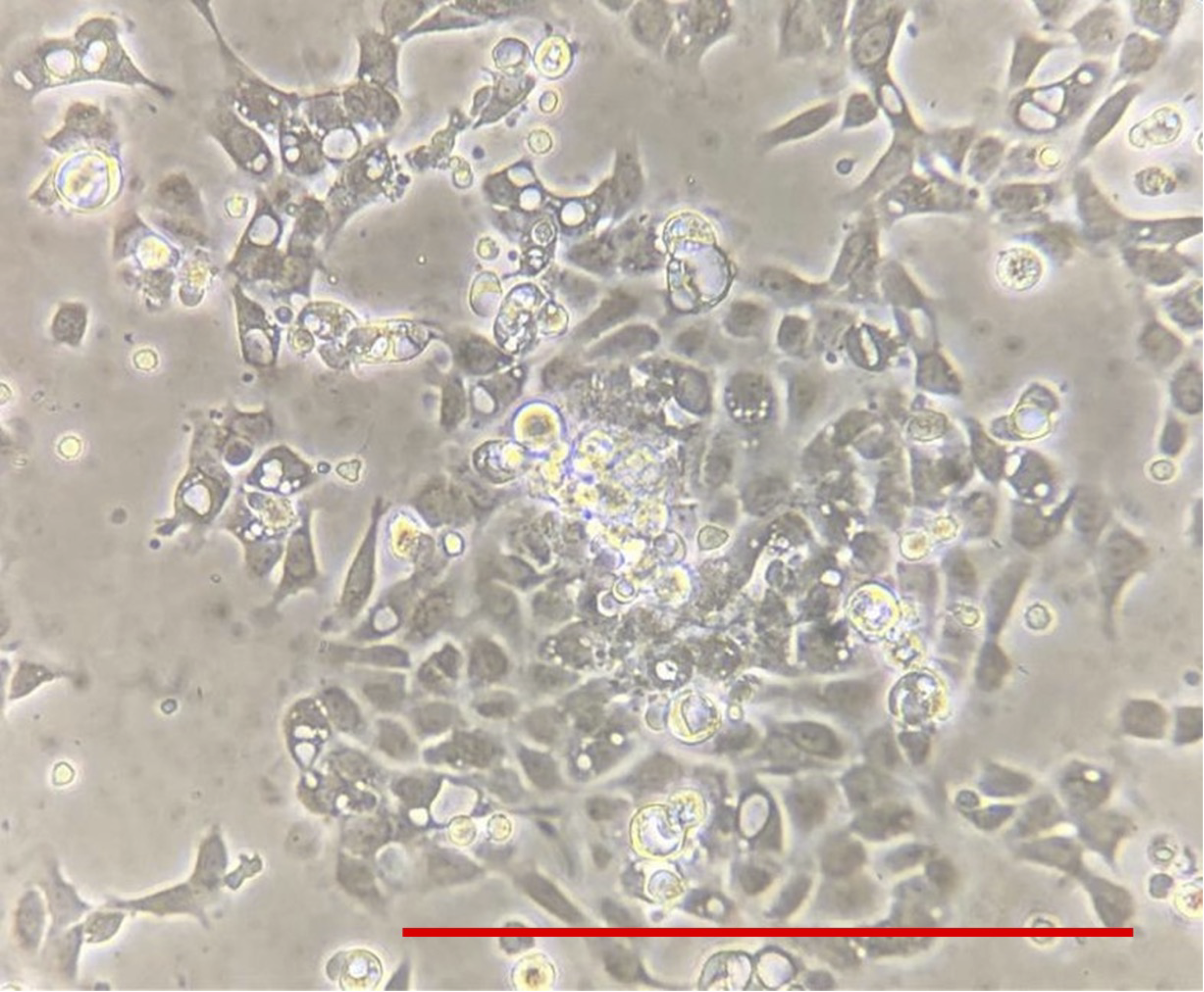

Supplement: Supplementary file 1 — Supplementary file1 Morphology of TMPCs after 8 h in culture flask, 40 × magnification, scale bar: 100 µm. (PNG 10703 KB) [file 44164_2024_79_MOESM1_ESM.png]

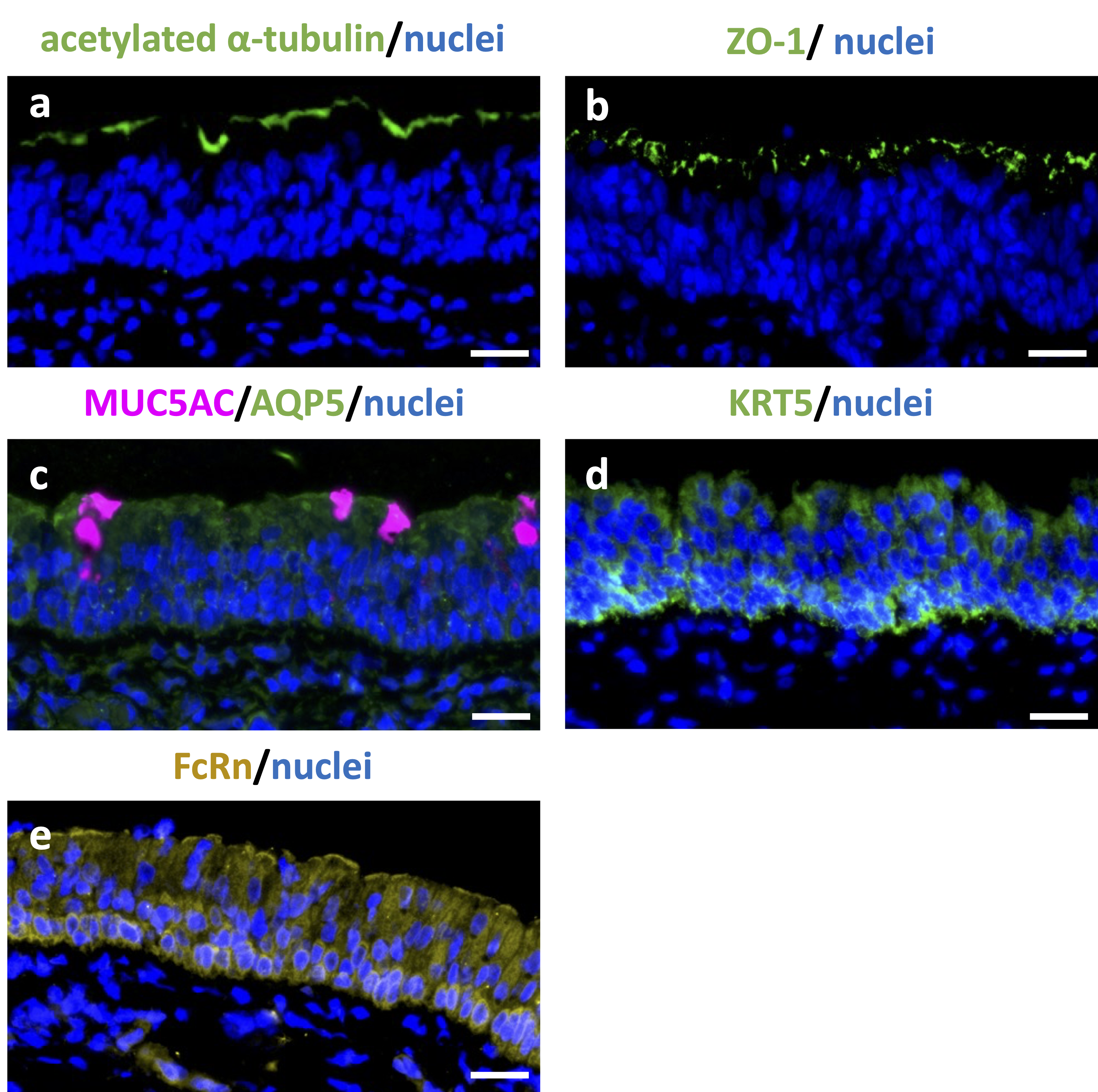

Supplement: Supplementary file 2 — Supplementary file2 Representative images of immunoreactivities against several characteristics of epithelial cells with nuclei counterstained with DAPI in blue for tracheal tissue: (a) acetylated tubulin as component of cilia is organized in hairy like structures. (b) ZO-1 indicative for the formation of tight junctions was observed in the typical apical location in the epithelium. (c) Mucus-secreting cells were identified by immunoreactivity against the mucin MUC5AC as well as against the water channel aquaporin-5 (AQP5). (d) Cytokeratin 5 (KRT5) as marker for basal cells was detected mainly in the basal cell layer of the epithelium. (e) Uptake of IgG was recently demonstrated to be mediated in the airway mucosa via FcRn, and comparable with the qPCR data, the epithelium showed a robust immunoreactivity against FcRn. All images were taken using a 40-fold air objective. All scale bars: 20 µm. (PNG 5843 KB) [file 44164_2024_79_MOESM2_ESM.png]

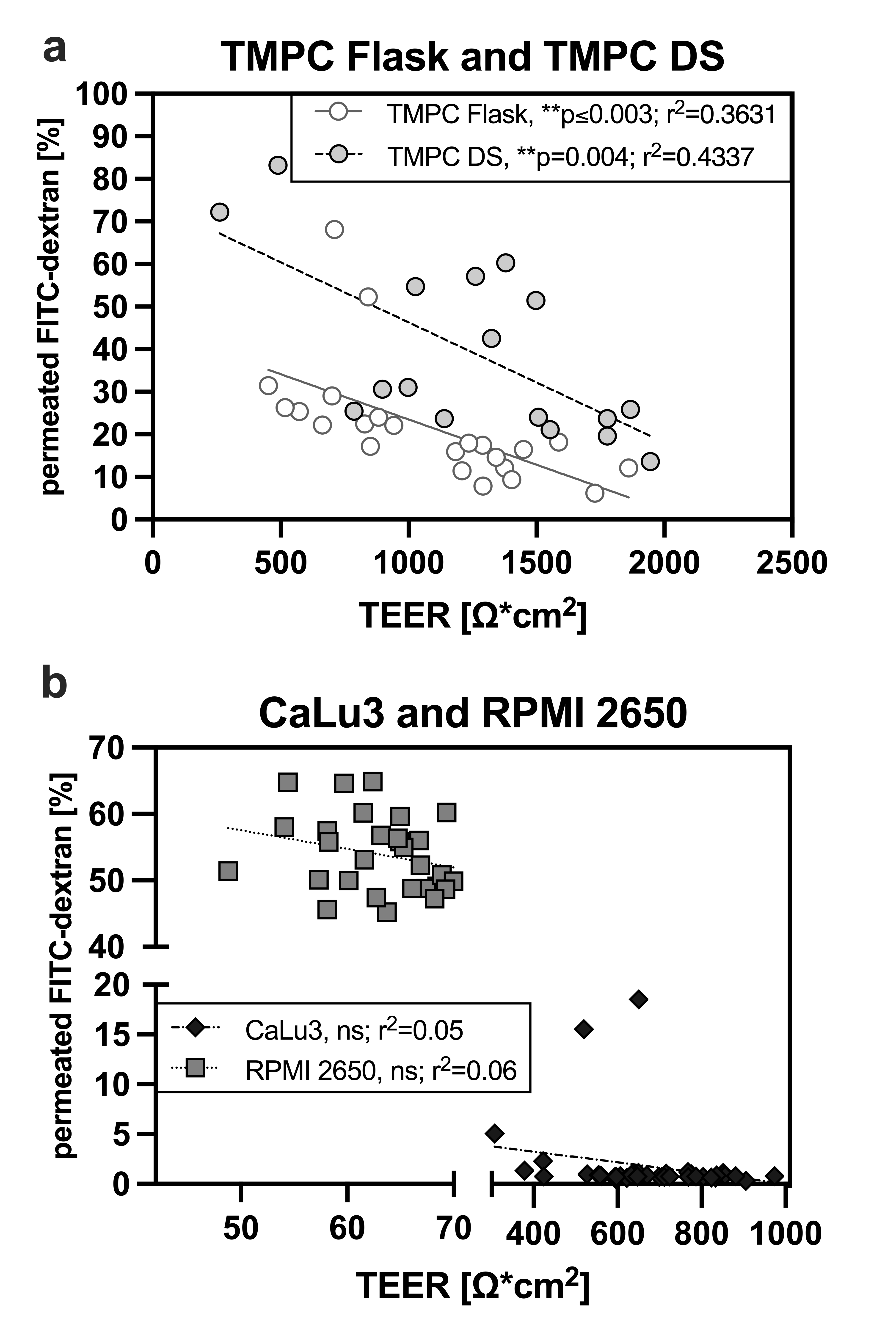

Supplement: Supplementary file 4 — Supplementary file4 Correlation of FITC – dextran permeation after 24 h with initial TEER values for different cell culture models after 21 days differentiation under ALI cultivation. (A) A significant correlation was observed for TMPC DS and TMPC Flask while (B) no correlation was found for CaLu3 and RPMI 2650. TMPC DS: n = 23, N = 1; TMPC Flask, n = 23, N = 1. B RPMI 2650: n = 30, N = 1; CaLu3: n = 36, N = 2; ns, not significant. (PNG 1147 KB) [file 44164_2024_79_MOESM4_ESM.png]
